# The Potential of Dark Septate Endophytes to Form Root Symbioses with Ectomycorrhizal and Ericoid Mycorrhizal Middle European Forest Plants

**DOI:** 10.1371/journal.pone.0124752

**Published:** 2015-04-23

**Authors:** Tereza Lukešová, Petr Kohout, Tomáš Větrovský, Martin Vohník

**Affiliations:** 1 Department of Plant Experimental Biology, Faculty of Science, Charles University in Prague, Prague, Czech Republic; 2 Department of Mycorrhizal Symbioses, Institute of Botany ASCR, Průhonice, Czech Republic; 3 Department of Botany, Institute of Ecology and Earth Sciences, University of Tartu, Tartu, Estonia; 4 Laboratory of Environmental Microbiology, Institute of Microbiology ASCR, Prague, Czech Republic; Institute for Sustainable Plant Protection, C.N.R., ITALY

## Abstract

The unresolved ecophysiological significance of Dark Septate Endophytes (DSE) may be in part due to existence of morphologically indistinguishable cryptic species in the most common *Phialocephala fortinii* s. l.—*Acephala applanata* species complex (PAC). We inoculated three middle European forest plants (European blueberry, Norway spruce and silver birch) with 16 strains of eight PAC cryptic species and other DSE and ectomycorrhizal/ericoid mycorrhizal fungi and focused on intraradical structures possibly representing interfaces for plant-fungus nutrient transfer and on host growth response. The PAC species *Acephala applanata* simultaneously formed structures resembling ericoid mycorrhiza (ErM) and DSE microsclerotia in blueberry. *A*. *macrosclerotiorum*, a close relative to PAC, formed ectomycorrhizae with spruce but not with birch, and structures resembling ErM in blueberry. *Phialocephala glacialis*, another close relative to PAC, formed structures resembling ErM in blueberry. In blueberry, six PAC strains significantly decreased dry shoot biomass compared to ErM control. In birch, one *A*. *macrosclerotiorum* strain increased root biomass and the other shoot biomass in comparison with non-inoculated control. The dual mycorrhizal ability of *A*. *macrosclerotiorum* suggested that it may form mycorrhizal links between Ericaceae and Pinaceae. However, we were unable to detect this species in Ericaceae roots growing in a forest with presence of *A*. *macrosclerotiorum* ectomycorrhizae. Nevertheless, the diversity of Ericaceae mycobionts was high (380 OTUs) with individual sites often dominated by hitherto unreported helotialean and chaetothyrialean/verrucarialean species; in contrast, typical ErM fungi were either absent or low in abundance. Some DSE apparently have a potential to form mycorrhizae with typical middle European forest plants. However, except *A*. *applanata*, the tested representatives of all hitherto described PAC cryptic species formed typical DSE colonization without specific structures necessary for mycorrhizal nutrient transport. *A*. *macrosclerotiorum* forms ectomycorrhiza with conifers but not with broadleaves and probably does not form common mycorrhizal networks between conifers with Ericaceae.

## Introduction

Fungal endophytes are defined as mycobionts which live inside living plant tissues, lack localized interfaces or specialized hyphae for nutrient transfer, their development is not synchronized with plant development and the plant does not nutritionally benefit from the symbiosis [1]. One of the most studied groups of fungal root endophytes, the so-called Dark Septate Endophytes (DSE), are a polyphyletic aggregate of fungi belonging to Class 4 of non-clavicipitaceous endophytes [2] which is broadly defined by the endophytic life strategy and presence of intraradical dark septate hyphae. The arguably most studied group of DSE is the *Phialocephala fortinii* s. l.—*Acephala applanata* species complex (PAC) [3] which comprises fungi that were formerly regarded as a single species, *Phialocephala fortinii* Wang & Wilcox (Helotiales, Ascomycota) [4]. However, it has been recently shown that this taxon is composed of at least 21 reproductively isolated lineages representing putative cryptic PAC species, out of which 8 were formally described [5]. Endophytes related to *P*. *fortinii* s. l. show low to any host preference [6,7], they are common inhabitants of both mycorrhizal and non-mycorrhizal roots and frequently live as endophytes in roots of conifers, ericaceous plants and orchids in both the Northern and Southern Hemispheres [8–10]. Although ubiquitous on dry land, PAC species seem to be less frequent to absent in roots of aquatic plants [11] which are probably colonized by other guilds of fungal endophytes [12]. Despite their frequent occurrence in terrestrial roots [6] and long coexistence with plants [13], the DSE influence on their hosts is still under debate. Results of numerous re-synthesis experiments are inconsistent—some report positive or neutral effects on plant growth [14–17] while others indicate mostly negative outcomes [18–20]. A recent meta-analysis of 18 research articles identified only positive influence of inoculation with DSE [21]. In contrast, another meta-analysis suggested negative to neutral effects of inoculation with non-clavicipitaceous root fungal endophytes (including DSE) on host plant biomass and nitrogen content [22].

Because most DSE are thought to lack specialized interfaces for nutrient transfer with their plant partners, the cause of the reported positive influence of inoculation with DSE remains unclear. Possible explanations include mineralization of the substrate that can be then exploited by the host roots [23], similarly to non-symbiotic saprotrophic fungi which may be even more effective in supplying nutrients to plants than mycorrhizal fungi [24]; production of phytohormones or analogous substances [25]; breaking down complex carbohydrates and providing simple sugars to the host plants, especially at the seedling stage [26]; reduction of disease intensity of some fungal pathogens, either active or passive [27]; or reduction of intraspecific competition between adult trees and their offspring [20]. However, many of these possible explanations remain at the level of hypotheses which need to be thoroughly tested under realistic experimental conditions. On the other hand, the number of structural studies investigating DSE associations in plant roots is considerably low [28].

DSE colonization is characterized by formation of microsleclerotia—aggregations of irregularly lobed hyphae [29] and dark septate hyphae growing inter- and intracellulary in the host root. In contrast, only a few DSE species were reported to form intraradical structures resembling those formed in mycorrhizal symbioses. For example, *P*. *fortinii* s. s. formed loose intracellular hyphal loops morphologically resembling ericoid mycorrhizae in a *Rhododendron* cultivar [30], *Heteroconium chaetospira* (Grove) M. B. Ellis formed similar structures in *Rhododendron obtusum* var. *kaempferi* (Planch.) Wilson [31] and *Acephala macrosclerotiorum* Münzenberger & Bubner formed a Hartig net and a hyphal mantle in axenic culture with Scots pine (*Pinus sylvestris* L.) [32]. Interestingly, some DSE seem to be able to simultaneously form structures resembling ericoid mycorrhizae and DSE colonization in the same ericaceous root [33]. However, functional aspects of these intraradical hyphal structures, i.e., nutrient transfer and/or plant growth response to colonization, are only rarely investigated [16,34] which is problematic especially in the case of Ericaceae mycobionts, because ericaceous rhizodermis can be colonized by a wide range of non-mycorrhizal fungi, including typical basidiomycetous saprobes [35].

Mycorrhizal plants may be interconnected by common mycorrhizal networks (CMNs) which may influence their establishment, diversity, competition and community dynamics [36]. Existence of interconnecting mycelia has been investigated for arbuscular mycorrhizal, ectomycorrhizal and ericoid mycorrhizal plants and their respective mycorrhizal fungi [37–40] but the role of fungal root endophytes remains unknown. A model mycorrhizal/DSE candidate potentially linking ectomycorrhizal conifers with ericoid mycorrhizal understorey might be ectomycorrhiza-forming *A*. *macrosclerotiorum* provided it forms ericoid mycorrhizae in Ericaceae at localities with the presence of its ectomycorrhizal morphotype *Pinirhiza sclerotia* [32]. However, to our knowledge, the ErM potential of *A*. *macrosclerotiorum* has not yet been investigated.

In this study we took an advantage of our culture collection of DSE strains which includes all hitherto described cryptic PAC species, namely *Acephala applanata*, *Phialocephala europaea*, *Phialocephala fortinii* s. s., *Phialocephala helvetica*, *Phialocephala letzii*, *Phialocephala subalpina*, *Phialocephala turiciensis* and *Phialocephala uotolensis* (authorities for all these species are Grünig & Sieber), together with other related DSE fungi (*A*. *macrosclerotiorum*, *Phialocephala glacialis* Grünig & Sieber), and tested the ability of different strains of these species to form specialized mycorrhizal structures in potentially ectomycorrhizal hosts (coniferous Norway spruce and broadleaved silver birch) and in a potentially ericoid mycorrhizal host (European blueberry). We described their respective colonization patterns in the roots of the host plants and compared these results with typical ectomycorrhizal and ericoid mycorrhizal fungi. The influence of inoculation with selected strains on plant growth under *in vitro* conditions was tested for blueberry and birch. Additionally, we investigated whether *A*. *macrosclerotiorum* is present in ericaceous hair roots in a natural Scots pine forest with occurrence of the ectomycorrhizal morphotype *P*. *sclerotia*, using both mycobiont isolation into pure culture and tag-encoded pyrosequencing, to tackle the possible mycelial/mycorrhizal links between ectomycorrhizal pines and their ericoid mycorrhizal understory.

## Materials and Methods

### Plant material

Norway spruce [*Picea abies* (L.) Karst.; spruce in the following text] was chosen as a model plant because it is arguably the most common forest conifer species in Central Europe. Both non-mycorrhizal and ectomycorrhizal spruce roots regularly host PAC [3,9]. European blueberry (*Vaccinium myrtillus* L.; blueberry) is arguably the most common forest ericaceous species in Central Europe which frequently co-occurs with conifers [41]. Silver birch (*Betula pendula* Roth; birch) is a common broadleaf species occurring across whole Central Europe and was included to explore the suggested specificity of *A*. *macrosclerotiorum* for conifers [32].

### Fungal material

We tested 10 different DSE species, each being represented by two different strains; 8 species belonged to PAC (*Acephala applanata*, *Phialocephala europaea*, *Phialocephala fortinii* s. s., *Phialocephala helvetica*, *Phialocephala letzii*, *Phialocephala subalpina*, *Phialocephala turiciensis*, *Phialocephala uotolensis*) and 2 species were related to but outside PAC (*Acephala macrosclerotiorum*, *Phialocephala glacialis*). One strain of *Paxillus involutus* (Batsch) Fr. was used as a positive ectomycorrhizal control and two strains of *Rhizoscyphus ericae* (Read) Zhuang & Korf as a positive ericoid mycorrhizal control (Table 1).

**Table 1:** Fungal strains investigated in this study.

Species/putative ecology	Strain Genbank no.	Source/country	Reference
***Acephala applanata*/**DSE (PAC)	AAP-1 EF093158	*Picea abies* root tip/Czech Republic	[9]
	AAP-2 n. a.	*Picea abies* root tip/Czech Republic	unpubl.
***Acephala macrosclerotiorum*/**DSE (outside PAC)	AMA-1 EU882732	*Pinus sylvestris* root tip/Germany	[32]
	AMA-11 n. a.	*Pinus sylvestris* root tip/Czech Republic	unpubl.
***Paxillus involutus***/ectomycorrhizal	PIN5 n.a.	Fruit body/Czech republic	unpubl.
***Phialocephala europaea*/**DSE (PAC)	PF-EU-1 JN091538	*Picea abies* root/Switzerland	[5]
	PF-EU-2 JN091540	*Picea abies* root/Switzerland	[5]
***Phialocephala fortinii* s. s./**DSE (PAC)	PFO-F EF446149	*Vaccinium myrtillus* hair root/Czech Republic	[9]
	PFO-9 n.a.	*Pinus sylvestris*/Czech Republic	unpubl.
***Phialocephala glacialis*/**DSE (outside PAC)	PF-GL-1 EU434843	*Vaccinium myrtillus* root/Switzerland	[70]
	PF-GL-2 EU434842	*Picea abies* needle/Switzerland	[70]
***Phialocephala helvetica*/**DSE (PAC)	PF-HE-1 JN091541	*Picea abies* root/Switzerland	[5]
	PF-HE-2 JN091543	*Picea abies* root/Switzerland	[5]
***Phialocephala letzii*/**DSE (PAC)	PF-LE-1 JN091534	*Picea abies* root/Switzerland	[5]
	PF-LE-2 JN091536	*Picea abies* root/Switzerland	[5]
***Phialocephala subalpina*/**DSE (PAC)	PF-SU-1 JN091551	*Vaccinium myrtillus* root/Switzerland	[5]
	PF-SU-2 JN091553	*Picea abies* root/Switzerland	[5]
***Phialocephala turiciensis*/**DSE (PAC)	PFO-2 EF093162	*Picea abies* root tip/Czech Republic	[9]
	PFO-6 EF093157	*Picea abies* root tip/Czech Republic	[9]
***Phialocephala uotolensis*/**DSE (PAC)	PF-UO-1 JN091547	*Picea abies* root/Switzerland	[5]
	PF-UO-2 JN091548	*Picea abies* root/Switzerland	[5]
***Rhizoscyphus ericae*/**ericoid mycorrhizal	RER-1 AJ319078	*Calluna vulgaris* hair root/UK	[82]
	RER-6 AF081437	*Calluna vulgaris* root/UK	[82]

DSE = dark septate endophyte; PAC = belonging to the *Phialocephala fortinii* s. l.—*Acephala applanata* species complex; n. a. = data not available.

### Experiment 1: *In vitro* inoculation of spruce and blueberry in an agar substrate

Blueberry and spruce seeds were surface sterilized in 30% H_2_O_2_ for 12 and 21 minutes, respectively and then rinsed twice in autoclaved deionized water. Spruce seeds were placed on the MMN medium [42] containing (NH_4_)_2_HPO_4_ 0.25 g, KH_2_PO_4_ 0.5 g, MgSO_4_.7H_2_O 0.15 g, CaCl2.2H_2_O 0.05 g, NaCl 0.025 g, FeEDTA 0.02 g, glucose 10.0 g, malt extract 3 g, thiamine 100 μg, agar 7.5 g, deionized water 1000 ml and germinated in Petri dishes (diam. 9 cm) at room temperature in the dark for 2 weeks. Blueberry seeds were germinated on water agar (agar 10 g, deionized water 1000 ml) in Petri dishes (diam. 9 cm) at room temperature in the dark for 2 weeks. All fungal strains listed in Table 1 except *P*. *involutus* were used for inoculation; they were pre-cultivated on MMN at room temperature in the dark.

Square Petri dishes (12 × 12 cm) were filled with the MMN medium without glucose and malt extract and left to cool down. Two thirds of the solidified media were then removed leaving approximately 40 ml of the media in the lower part of each dish. The solidified medium was then covered by an autoclaved cellulose foil and each dish was inoculated with 9 agar plugs dissected from the pre-cultivated fungal cultures (see above).

One spruce and two blueberry seedlings were inserted into each dish with their roots placed on the surface of the medium covered by the foil. To prevent desiccation the roots were covered by a moistened autoclaved filter paper. Folded autoclaved filter paper was inserted in between the lid and the bottom of the dish to enable gas exchange and the dish was then sealed with an air permeable foil. To shade the roots the bottom part of each dish was wrapped with an aluminum foil. The dishes were stored vertically in a growth chamber (16 hours of light at 20C, one tungsten lamp and relative humidity 80%; Fitotron, SANYO, UK). There were three dishes per each inoculation treatment.

One dish per treatment was harvested after 3 months, the other two dishes one month later. Roots were washed in tap water and prepared for screening as follows. Blueberry roots were placed in 10% KOH, autoclaved for 10 minutes, rinsed in tap water, acidified in 3% HCl and stained in a solution of trypan blue in lactoglycerol (1: 1: 3 v.v. lactic acid: glycerol: deionized water, 0.05% solution) and then de-stained in lactoglycerol. An upright Olympus BX-60 microscope with differential interference contrast at high magnifications (400× and 1000×) was used to observe inter- and intracellular hyphal structures. Intact spruce roots removed from the dishes were observed under a dissecting microscope, ectomycorrhizal and non-colonized root tips were counted and the total root length was measured. One or two colonized root tips per each strain were cross-sectioned using a razor blade. Cross-sections were then observed with the upright microscope at high magnifications (400× and 1000×).

### Experiment 2: *In vitro* inoculation of blueberry in a peat-based substrate

This experiment was set up to further investigate the influence of DSE on blueberry. However, due to low biomass and high mortality of blueberry seedlings in the previous experiment we decided to use different cultivation design, similar to that used by Vohník *et al*. [35]. In brief, both blueberry seedlings and fungal inoculum were pre-cultivated as described above. 10 ml of autoclaved MMN without sugars was poured into 50 ml sterile plastic tubes and left to solidify. One agar plug (diam. 0.5 cm) was dissected from a margin of an actively growing colony of the selected fungal strains (Table 1, the same strains as in Experiment 1) and transferred to the surface of the medium in each tube. Tubes for control plants were inoculated with agar plugs without fungal mycelium. The tubes were incubated in the dark at room temperature and the growth of the mycelium was checked periodically. After two months, 10 ml of twice autoclaved peat (pH before and after autoclaving 3.9 and 4.0, respectively) were added to the tubes which were then incubated in the dark at room temperature for another two weeks to enable fungal colonization of the peat. Single two-months-old sterile blueberry seedling were then inserted into the tubes which were consequently closed by lids, sealed with an air permeable film and incubated in the growth chamber under the same regime as above. The seedlings were harvested after 3.5 months. Their roots were separated from shoots, gently washed under running tap water, dried with towel paper and weighted. The roots were then stained and de-stained as above. The percentage of colonized rhizodermal cells was measured using the upright microscope at 400× magnification as a proportion of 500 randomly screened rhizodermal cells per root system. The shoots were dried (90 min at 65°C) and weighted. Each treatment including non-inoculated control had 6 tubes (= replications).

### Experiment 3: *In vitro* inoculation of birch in a peat-agar medium

In this experiment we tested whether *A*. *macrosclerotiorum* was able to form ectomycorrhizae in roots of a broadleaved tree under *in vitro* conditions, similarly to Münzenberger *et al*. [32]. Birch seeds were surface sterilized in 30% H_2_O_2_ for 12 minutes and rinsed twice in autoclaved tap water. They were germinated in Petri dishes (diam. 9 cm) filled with MMN without glucose and malt extract at room temperature in the dark for three weeks, the seedlings were then cultivated for another four weeks in the growth chamber under the regime described above. Four fungal strains were selected for this experiment: two strains of *A*. *macrosclerotiorum* (AMA-1 and AMA-11), one strain of *A*. *applanata* (AAP-1) as a representative of PAC and one strain of *P*. *involutus* (PIN-5) as a representative of ectomycorrhizal fungi (Table 1). The experimental scheme was similar to Experiment 2: agar plugs with fungal mycelium were inserted into falcon tubes with 10 ml of solid MMN without glucose and malt extract, cultivated for one month in the dark and covered with 10 ml of a twice autoclaved peat-vermiculite substrate (volume ratio 1: 5: 6 peat: vermiculite: water). One birch seedling was then planted into each tube which was then closed, sealed and incubated in the growth chamber under the same regime as described above. Each treatment including control had 6 tubes (= replications).

Three days prior the harvest CO_2_ concentration was measured using an HP 6850 gas chromatograph (Agilent, USA) equipped with a 0.53 mm × 15 m HP-Plot Q column and a 0.53 mm ×15 m HP-Plot Molecular Sieve 5A column, and a thermal conductivity detector, using helium as a carrier gas.

The seedlings were harvested after 6 months. Their roots were separated from shoots, gently washed with running tap water, surface dried with towel paper, weighted, stained with trypan blue in lactoglycerol, then de-stained in lactoglycerol (see above). The de-stained roots were screened using the upright microscope at high magnifications (400× and 1000×) and presence of microsclerotia and intraradical hyphae was counted in 80 microscopic fields per each root system under 400× magnification [11]. Colonization was not quantified in positive control (plants inoculated with *P*. *involutus*) but presence of Hartig net was checked to see whether the cultivation conditions were favorable for formation of ectomycorrhizal symbiosis. Shoots were dried (90 min at 65°C) and weighted. To check for contaminations and viability of the inoculated fungi four samples of the peat substrate were aseptically taken from each of the microcosms and placed on MMN media in Petri dishes (diam. 9 cm). The dishes were kept for one month in the dark at 20°C and periodically screened for signs of fungal growth.

### Statistical analyses

The data obtained in Experiments 2 and 3 were analyzed using the STATISTICA 12 software (StatSoft Inc., USA). As they did not meet the ANOVA criteria for normal distribution and homogeneity of variances the non-parametric Kruskal-Wallis test followed by a multiple-comparison z-value test were used. Correlation between colonization and root fresh weight and shoot dry weight for inoculated plants in Experiment 2 was tested using Spearman R correlation.

### Culture dependent screening of DSE-like fungi in Ericaceae roots from a pine forest

Ten soil cores (diam. 12 cm) from under Ericaceae (European blueberry) shrubs were collected in Národní Park České Švýcarsko (= Bohemian Switzerland National Park) at the site CS1 (N 50°52.190´, E 14°22.903´; 310 m a. s. l.) in June 2011. The sampling was performed in accordance with a sampling permit issued by the Bohemian Switzerland National Park Authority (contract #SNPCS 02084/2011, permit #134/2011). The site is dominated by *P*. *sylvestris* forest with understory of ericaceous plants [*V*. *myrtillus*, *Vaccinium vitis-idaea* L. and *Calluna vulgaris* (L.) Hull] on podzolic soil. Ericaceae roots were washed under a tap water and separated into two parts: the first part was used for measurement of fungal colonization (as above), the second part for mycobiont isolation. Two hundred and forty randomly selected root pieces (approx. 5 mm long) were surface sterilized 30 s in 10% SAVO (a trade name of a common Czech household bleach contains 4.5% available chlorine; Unilever ČR Ltd., Czech Republic), rinsed twice in deionized water and plated on MMN with lowered concentration of glucose (1g per liter) in Petri dishes (diam. 9 cm). The dishes were sealed with an air-permeable tape and incubated in the dark at 20°C for one month. DNA was extracted from all darkly pigmented mycelia using REDExtract-N-Amp PCR kit (Sigma-Aldrich, Germany) following manufacturer’s instructions. Four μl of isolated DNA were further amplified using the fungal specific primer pair ITS1F/ITS4. The PCR products were purified and sequenced at Macrogen Europe Laboratory (Macrogen Inc., South Korea). The obtained sequences were screened in Finch TV v1.4.0 (geospiza.com/finchtv), edited when needed and subjected to BLAST searches (megablast/blastn algorithms) in GenBank [43] to find out whether they comprise sequences of *A*. *macrosclerotiorum*. For species identification, at least three closest matches preferably derived from cultured fungi with scientific names were considered and their taxonomic position was further checked with Blast Tree View (NJ, max. seq. difference 0.75).

### Culture independent screening of fungal root symbionts in Ericaceae roots from the pine forest

Fifteen samples were collected at three different experimental sites [CS1 (as above), CS2 (N 50°52.719´, E 14°22.806´) and CS3 (N 50°52.387´, E 14°26.725´)] in NP České Švýcarsko in September 2011 in accordance with the sampling permit (see above). The root samples were taken by a soil corer (diam. 5 cm x 10 cm) from patches overgrown by blueberry shrubs. The samples were then washed with tap water, Ericaceae and pine roots were separated and the pine roots were observed under a dissecting microscope for presence of the *P*. *sclerotia* morphotype. When *P*. *sclerotia* was present, neighboring Ericaceae roots were surface sterilized in 30 s in 30% H_2_O_2_, rinsed twice in autoclaved deionised water, surface-dried with a paper towel and placed in sterile Eppendorf tubes in a freezer at -20°C. In this way we selected five samples from CS1, three from CS2 and two from CS3.

The roots were weighted and samples weighing between 50 and 100 μg were prepared. The roots were then homogenized in liquid nitrogen. DNA was extracted using the DNeasy Plant Mini kit (QIAGEN, Germany) following manufacturer´s instructions. Isolated DNA was 10x diluted in dd H_2_O and amplified in 4 independent PCR reactions using primers ITS1F and ITS4. Each PCR mix consisted of 16.75 μl ddH_2_O, 2.5 μl 10x Taq PCR buffer with KCl (Thermo Scientific, USA), 2 μl MgCl_2_ (25 mM), 0.5 μl dNTP mixture (2 mM each), 0.5 μl of each primer (10 μM), 1 μl BSA (20 mg/ml; Sigma, USA), 1U Taq DNA polymerase (Thermo Scientific, USA) and 2 μl of diluted DNA template. Vapo.protect Mastercycler (Eppendorf, Germany) was used with following parameters: 94°C for 4 min, then 24 cycles at 94°C for 30 s, 55°C for 30 s, and 72°C for 90 s followed by 72°C for 10 min. To increase concentration of DNA the PCR product was purified using a QIAquick PCR purification kit (QIAGEN, Germany). To clear the sample from primers from the first PCR a Zymoclean GEL DNA Recovery kit (ZYMO RESEARCH, USA) was used.

After purification PCR with tagged primers was performed. PCR mix consisted of 31 μl ddH_2_O, 10 μl PCR buffer with MgCl_2_ (5x Phusion HF buffer containing 7.5 mM MgCl_2_; Thermo Scientific, USA), 1.5 μl DMSO (dimethyl sulfoxide; Sigma, USA), 1 μl dNTP mixture (2 mM each), 0.5 μl tag-ITS 1 and tag-ITS 4 primers (10 μM), 0.2 μl Phusion polymerasis (2000 U/ml, BioLabs New England, USA) and 1 μl of template. The cycler settings were 94°C for 5 min, then 20 cycles at 94°C for 30 s, 55°C for 30 s, and 72°C for 90 s followed by 72°C for 10 min. The resulting PCR products were purified using the QIAquick PCR purification kit and the Zymoclean GEL DNA Recovery kit. Purified products were subsequently equimolarly mixed and pyrosequenced in GATC Biotech (Konstanz, Germany) using the Roche GS FLX+ platform.

In total, the tag-encoded pyrosequencing yielded 33999 raw sequences. The data were filtered and trimmed using the pipeline SEED ver. 1.2.1 [44]. All sequences with mismatches in tags were removed from the dataset. A pyrosequencing noise reduction was performed using the PyroNoise algorithm translation in Mothur ver. 1.28.0 [45]. Chimeric sequences were detected using Uchime implementation in USEARCH ver. 7.0.1090 [46] and deleted. A total of 22653 sequences were retained after the removal of low-quality sequences (mean < 25), sequences shorter than 480 bases and potentially chimeric sequences. This dataset was trimmed to the 480 bp sequence length and the ITS1 region was extracted using ITSx [47]. This yielded 22634 extracted ITS1 sequences which were clustered to OTUs using UPARSE implementation in USEARCH ver. 7.0.1090 [48] with 97% similarity threshold (794 chimeric sequences were excluded during this step). The consensus sequence from each OTU was constructed from a MAFFT alignment [49] based on the most abundant nucleotide at each position.

For sample comparison and diversity analyses the dataset was randomly resampled at the same sampling depth of 325 sequences. Diversity indices were computed in SEED ver. 1.2.1. OTUs identification was performed by BLASTn against local ITS database derived from NCBI GenBank on 10th March 2014. Each OTU was assigned at the taxonomic level of order (or nearest lower or higher level when order was not specified) by comparing the BLASTn best hits with the taxonomic information derived from the NCBI taxonomy server. Principal component analysis (PCA) was performed using PAST ver. 2.17c taking into account the number of sequences obtained for each OTU [50] and custom edited.

The taxonomic positions of OTU 1, OTU 4 and all OTUs related to the *Rhizoscyphus ericae* aggregate were further checked by BLAST Tree View using BLAST pairwise alignments (NJ, max. seq. difference 0.75). The closest BlastN matches to OTU1 and OTU4 sequences were downloaded from NCBI Genbank. Sequence alignments were obtained using MAFFT 6 (http://mafft.cbrc.jp/alignment/software) [51]. The final dataset with OTU1 sequences had 105 sequences and 166 characters and with OTU4 sequence had 111 sequences and 171 characters. Maximum likelihood (ML) analyses were performed in PhyML 3.0 [52], using Kimura-2 parameter model and bootstrap support was obtained using 100 replicates. Evolutionary models were determined using MEGA 5.05 [53].

## Results

Here we aimed at testing the central hypothesis that the variability in the PAC relationship with their host plants, i.e., the type of the relationship (mycorrhizal vs. endophytic vs. parasitic) and the resulting host plant growth response (positive vs. neutral vs. negative) is primarily cryptic species-dependent. The finding that *A*. *macrosclerotiorum* was able to form both ectomycorrhiza with spruce and ericoid mycorrhiza with blueberry *in vitro*, as well as the fact that it fromed ectomycorrhizae with pine in the screened forest, led us to hypothesize that this DSE species might mediate mycorrhizal interactions between conifers and their ericaceous undergrowth. We therefore tested the main prerequisite for this hypothesis, i.e., whether *A*. *macrosclerotiorum* occurred in neighboring conifer and Ericaceae roots under natural conditions. This additionally yielded the first comprehensive information on the spectra of mycobionts inhabiting hair roots of Ericaceae in central Europe based on tag-encoded pyrosequencing.

### Experiment 1: *In vitro* inoculation of spruce and blueberry in an agar substrate

**Fig 1:**
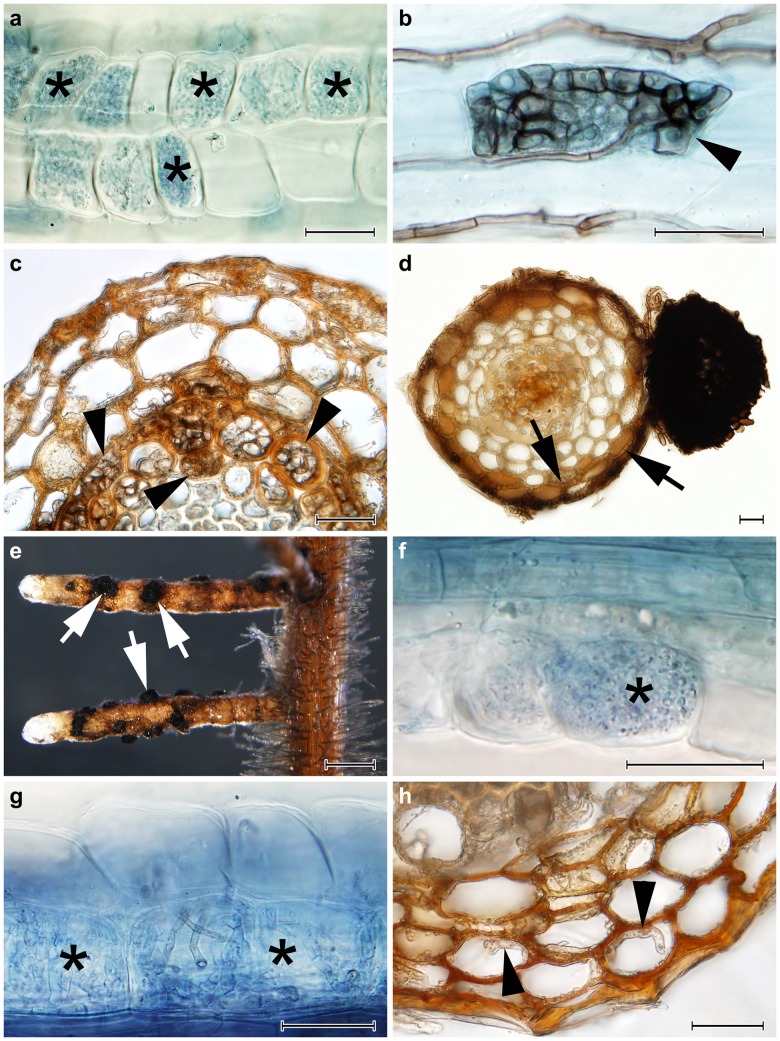
The colonization patterns observed in Norway spruce (*Picea abies*) and European blueberry (*Vaccinium myrtillus*) roots in Experiment 1. **1a)** Typical ericoid mycorrhizal colonization formed by *Rhizoscyphus ericae* in blueberry roots (asterisks); stained with trypan blue, observed with DIC, bar = 25 μm. **1b)** An intracellular microsclerotium formed by *Phialocephala helvetica* in a blueberry root (arrowhead); stained with trypan blue, observed with DIC, bar = 25 μm. **1c**) Intracellular microsclerotia formed by *P*. *helvetica* in the vascular cylinder of a spruce root (arrowheads); observed with DIC, bar = 25 μm. **1d)** A Hartig net formed within the spruce root cortex (arrows) and an extraradical sclerotium formed on the spruce root surface (asterisk) by *Acephala macrosclerotiorum*; observed with DIC, bar = 25 μm. **1e)** Spruce root tips colonized by *A*. *macrosclerotiorum* with extraradical superficial sclerotia formed on the root surface (arrows); bar = 0.5 mm. **1f)** Intracellular hyphal loops morphologically resembling ericoid mycorrhizae (asterisks) formed by *A*. *macrosclerotiorum* in blueberry roots; stained with trypan blue, observed with DIC, bar = 25 μm. **1g)** Loose intracellular hyphal loops which may morphologically resemble ericoid mycorrhiza (asterisks) formed by *Phialocephala glacialis* in blueberry roots; stained with trypan blue, bar = 25 μm. **1h)** Intracellular colonization of spruce root cortex by *P*. *glacialis* (arrowheads); bar = 25 μm.

Roots of all inoculated plants possessed intraradical fungal colonization whereas the non-inoculated control plants were free of any fungal colonization. In blueberry, both strains of *R*. *ericae* formed dense intracellular hyphal coils typical for ErM (Fig 1a). All species belonging to PAC formed intracellular microsclerotia consisting of melanised or hyaline hyphae in both spruce and blueberry (Fig 1b and 1c). In spruce, microsclerotia were often found within the central stele (Fig 1c). *A*. *macrosclerotiorum* colonized spruce intercellulary and formed a Hartig net and a parenchymatous hyphal net on the root surface resembling a loose hyphal mantle (Fig 1d), and darkly pigmented sclerotia on the surface of some roots (Fig 1d and 1e). In contrast to the tested PAC species *A*. *macrosclerotiorum* never formed intracellular microsclerotia in spruce roots. On the other hand, intracellular hyphal coils resembling ErM (Fig 1f) together with intracellular microsclerotia typical for DSE were observed in blueberry roots colonized by *A*. *macrosclerotiorum*. A colonization pattern resembling ErM was formed also by *P*. *glacialis* (Fig 1g) which however did not form EcM structures in spruce but colonized its roots intracellulary, including microsclerotia (Fig 1h) (Table 2).

**Table 2:** Fungal structures observed in the roots of Norway spruce (*Picea abies*), silver birch *(Betula pendula)* and European blueberry (*Vaccinium myrtillus*) colonized by the tested fungal strains.

Inoculated fungi		*Picea abies*				*Betula pendula*					*Vaccinium myrtillus*			
Species	Strain	Superficial sclerotia	Microsclerotia	Hyphal mantles	Hartig net	Superficial sclerotia	Microsclerotia	Intermediate structures	Hyphal mantles	Hartig net	Superficial sclerotia	Microsclerotia	Intermediate structures	Ericoid mycorrhiza
***Acephala applanata***	AAP-1	-/n/n	+/n/n	-/n/n	-/n/n	n/n/+	n/n/+	n/n/+	n/n/-	n/n/-	-/+/n	+/+/n	+/+/n	-/+/n
	AAP-2	-/n/n	+/n/n	-/n/n	-/n/n	n	n	n	n	n	-/-/n	+/+/n	+/+/n	-/+/n
***Acephala macrosclerotiorum***	AMA-1	+/n/n	-/n/n	+/n/n	+/n/n	n/n/-	n/n/-	n/n/+	n/n/-	n/n/-	-/+/n	+/-/n	+/+/n	+/+/n
	AMA-11	+/n/n	-/n/n	+/n/n	+/n/n	n/n/-	n/n/-	n/n/+	n/n/-	n/n/-	-/+/n	+/-/n	+/+/n	+/+/n
***Paxillus involutus***	PIN-9	n	N	n	n	n/n/-	n/n/-	n/n/-	n/n/+	n/n/+	n	n	n	n
***Phialocephala europaea***	PF-EU-1	-/n/n	+/n/n	-/n/n	-/n/n	n	n	n	n	n	-/-/n	+/+/n	-/-/n	-/-/n
	PF-EU-2	-/n/n	+/n/n	-/n/n	-/n/n	n	n	n	n	n	-/-/n	+/+/n	-/-/n	-/-/n
***Phialocephala fortinii* s. s.**	PFO-F	-/n/n	+/n/n	-/n/n	-/n/n	n	n	n	n	n	-/-/n	+/+/n	-/-/n	-/-/n
	PFO-9	-/n/n	+/n/n	-/n/n	-/n/n	n	n	n	n	n	-/-/n	+/+/n	-/-/n	-/-/n
***Phialocephala Glacialis***	PF-GL-1	-/n/n	+/n/n	-/n/n	-/n/n	n	n	n	n	n	-/-/n	+/+/n	+/+/n	-/-/n
	PF-GL-2	-/n/n	+/n/n	-/n/n	-/n/n	n	n	n	n	n	-/-/n	+/+/n	+/+/n	-/-/n
***Phialocephala Helvetica***	PF-HE-1	-/n/n	+/n/n	-/n/n	-/n/n	n	n	n	n	n	-/-/n	+/+/n	-/-/n	-/-/n
	PF-HE-2	-/n/n	+/n/n	-/n/n	-/n/n	n	n	n	n	n	-/-/n	+/+/n	-/-/n	-/-/n
***Phialocephala Letzii***	PF-LE-1	-/n/n	+/n/n	-/n/n	-/n/n	n	n	n	n	n	-/-/n	+/+/n	-/-/n	-/-/n
	PF-LE-2	-/n/n	+/n/n	-/n/n	-/n/n	n	n	n	n	n	-/-/n	+/+/n	-/-/n	-/-/n
***Phialocephala subalpina***	PF-SU-1	-/n/n	+/n/n	-/n/n	-/n/n	n	n	n	n	n	-/-/n	+/+/n	-/-/n	-/-/n
	PF-SU-2	-/n/n	+/n/n	-/n/n	-/n/n	n	n	n	n	n	-/-/n	+/+/n	-/-/n	-/-/n
***Phialocephala turiciensis***	PFO-2	-/n/n	+/n/n	-/n/n	-/n/n	n	n	n	n	n	-/-/n	+/+/n	-/-/n	-/-/n
	PFO-6	-/n/n	+/n/n	-/n/n	-/n/n	n	n	n	n	n	-/-/n	+/+/n	-/-/n	-/-/n
***Phialocephala uotolensis***	PF-UO-1	-/n/n	+/n/n	-/n/n	-/n/n	n	n	n	n	n	-/-/n	+/+/n	-/-/n	-/-/n
	PF-UO-2	-/n/n	+/n/n	-/n/n	-/n/n	n	n	n	n	n	-/-/n	+/+/n	-/-/n	-/-/n
***Rhizoscyphus Ericae***	RER-1	-/n/n	-/n/n	-/n/n	-/n/n	n	n	n	n	n	-/-/n	-/-/n	-/-/n	+/+/n
	RER-6	-/n/n	-/n/n	-/n/n	-/n/n	n	n	n	n	n	-/-/n	-/-/n	-/-/n	+/+/n

**S** = extraradical superficial sclerotia; **M** = intracellular microsclerotia; **Hy** = hyphal mantles; **Ha** = intercellular Hartig net; **I** = intermediate structures, i.e., darkly pigmented or hyline loose intracellular hyphal loops; **E** = intracellular hyphal coils similar or identical to those formed in ericoid mycorrhiza. “**+/-**” denotes presence/absence of the structure in the root sample and **n** denotes not tested. Data from different experiments are separated by slashes (Experiment 1/Experiment 2/Experiment 3).

### Experiment 2: *In vitro* inoculation of blueberry in a peat-based substrate

**Fig 2:**
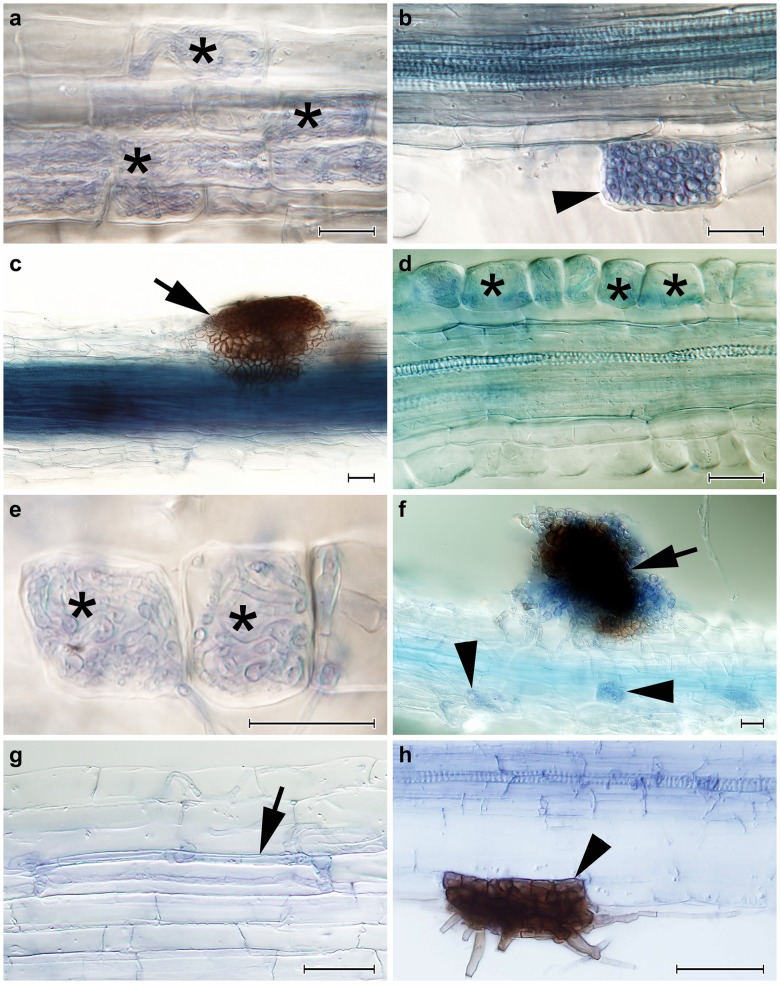
The colonization patterns observed in European blueberry (*Vaccinium myrtillus*) roots in Experiment 2 and in silver birch in Experiment 3. **2a)** Intracellular hyphal colonization resembling ericoid mycorrhiza formed by *Acephala applanta* AAP-1 in blueberry roots (asterisks). **2b)** An early stage of the development of an intracellular microsclerotium formed by *A*. *applanata* AAP-1 in a blueberry root (arrowhead). **2c)** An extraradical sclerotium formed on the surface of a blueberry root by *A*. *applanata* AAP-1 (arrow). **2d)** A blueberry hair root colonized in a manner resembling ericoid mycorrhiza by *Acephala macrosclerotiorum* AMA-11 (asterisks). **2e)** A detail of two blueberry rhizodermal cells intracellularly colonized by *A*. *macrosclerotiorum* AMA-11 in a manner resembling ericoid mycorrhiza (asterisks). **2f)** An extraradical sclerotium formed on the surface of a blueberry root by *A*. *macrosclerotiorum* AMA-11 (arrow). Note accompanying intracellular hyphal colonization (arrowheads). **2g)** A loose intracellular hyphal loop formed by *A*. *macrosclerotiorum* AMA-1 in a birch root (arrow). **2h)** A melanised intracellular microsclerotium formed by *Acephala applanata* AAP-1 in birch (arrowhead). All figures stained with trypan blue, observed with DIC, bars = 25 μm.

All of the inoculated blueberry seedlings possessed intraradical hyphal colonization similar to that found in Experiment 1 (Fig 2). Typical ErM structures were regularly formed in the roots of the plants inoculated with *R*. *ericae*. Intracellular hyphal coils resembling ErM colonization were formed by *A*. *applanata* (Fig 2a) which also formed intracellular microsclerotia and extraradical superficial sclerotia (Fig 2b and 2c, respectively), *A*. *macrosclerotiorum* (Fig 2d and 2e) which also formed extraradical superficial sclerotia (Fig 2f) and to some extent also by *P*. *glacialis*. All DSE strains except *A*. *macrosclerotiorum* formed intracellular melanised or hyaline microsclerotia. In contrast to Experiment 1 melanised sclerotia were formed on the root surface of the blueberry seedlings inoculated with *A*. *applanata* AAP-1 and both strains of *A*. *macrosclerotiorum* (see above; Table 2). Control plants did not possess any fungal colonization.

**Fig 3:**
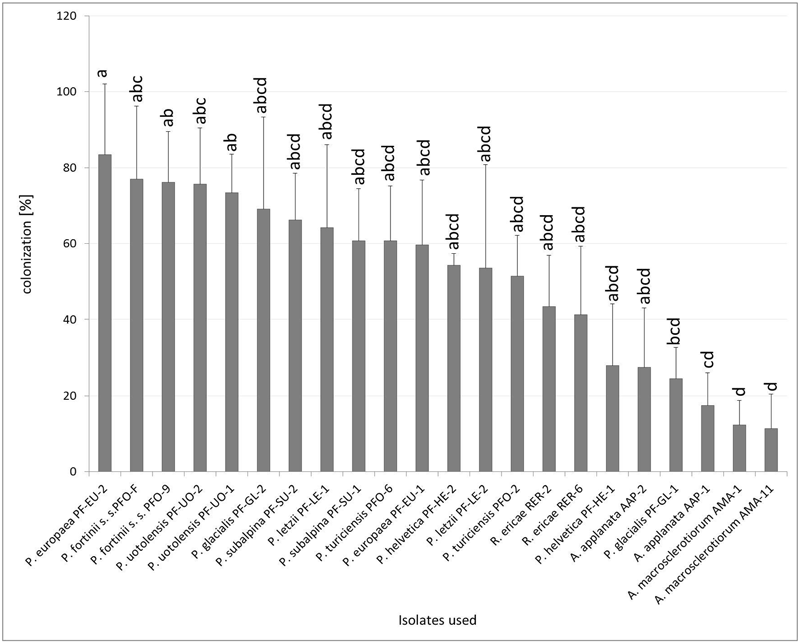
Percentage fungal colonization of blueberry rhizodermal cells in Experiment 2. Blueberry seedlings were inoculated by 8 PAC species, 2 species related to PAC and *Rhizoscyphus ericae* as a positive control, two strains per each species. Blueberry seedlings were grown in a peat-based substrate for 3.5 months under *in vitro* conditions. The presented data are means of 6 replicates ± standard error of mean. Different letters above the columns indicate significant differences according to the non-parametric Kruskal-Wallis test followed by the multiple-comparison z-value test.

The colonization rates varied among the tested strains (Fig 3). Some DSE strains that formed ErM-like structures in the roots—*A*. *macrosclerotiorum*, *A*. *applanata* AAP-1 and *P*. *glacialis* PF-GL-1—had significantly lower colonization rates than *P*. *europaea* PF-EU-2. Both strains of *A*. *macrosclerotiorum* had significantly lower colonization rates than both strains of *P*. *fortinii* s. s. and *P*. *uotolensis* but no other differences between this ErM forming fungus and PAC were found. The percentage of colonized root cells by *A*. *applanata* AAP-1 was significantly lower than *P*. *fortinii* s. s. PFO-9 and *P*. *uotolensis* PF-UO-1 but no other statistically significant differences within PAC were found. Apparent trends of intraspecific differences were observed especially in *P*. *glacialis* and *P*. *helvetica* although these were statistically non-significant (Fig 3).

**Fig 4:**
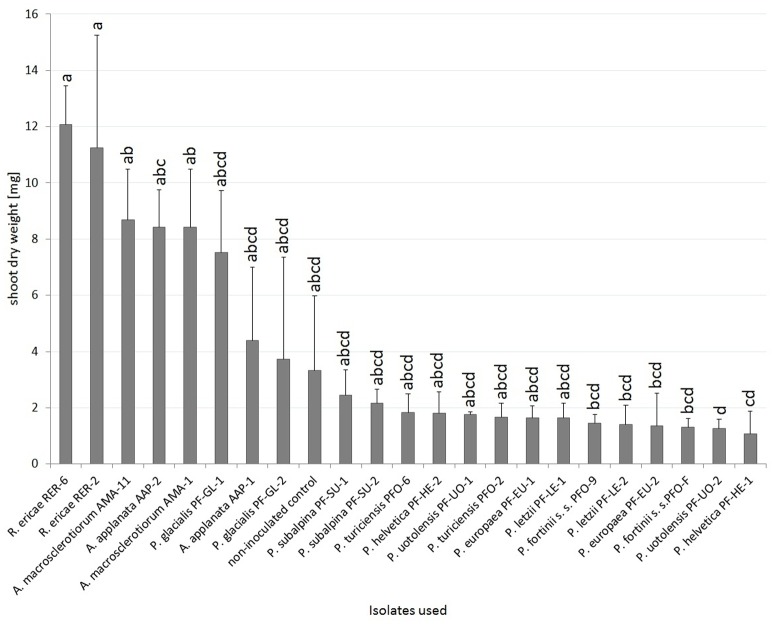
The effect of inoculation on blueberry dry shoot weight in Experiment 2. Blueberry seedlings were inoculated by 8 PAC species, 2 species related to PAC and *Rhizoscyphus ericae* as a positive control, two strains per each species. Blueberry seedlings were grown in a peat-based substrate for 3.5 months under *in vitro* conditions. The presented data are means of 6 replicates ± standard error of mean. Different letters above the columns indicate significant differences according to the non-parametric Kruskal-Wallis test followed by the multiple-comparison z-value test.

With respect to the influence of the inoculation on blueberry, six PAC strains (both *P*. *fortinii* s. s. strains, *P*. *europaea* PF-EU-2, *P*. *helvetica* PF-HE-1, *P*. *letzii* PF-LE-2 and *P*. *uotolensis* PF-UO-2) significantly decreased its dry shoot biomass when compared to positive mycorrhizal control inoculated with *R*. *ericae*. Blueberries inoculated with *A*. *applanata* AAP-2 had significantly higher dry shoot biomass than those inoculated with *P*. *uotolensis* PF-UO-2 but no other differences within PAC were found. Blueberries inoculated with both *A*. *macrosclerotiorum* strains showed higher dry shoot biomass than two PAC strains (*P*. *helvetica* PF-HE-1and *P*. *uotolensis* PF-UO-2). The highest (although statistically non-significant) growth differences between conspecific strains were observed in *A*. *applanata* (AAP-1 vs. AAP-2) and *P*. *glacialis* (PF-GL-1 vs. PF-GL-2) (Fig 4).

**Fig 5:**
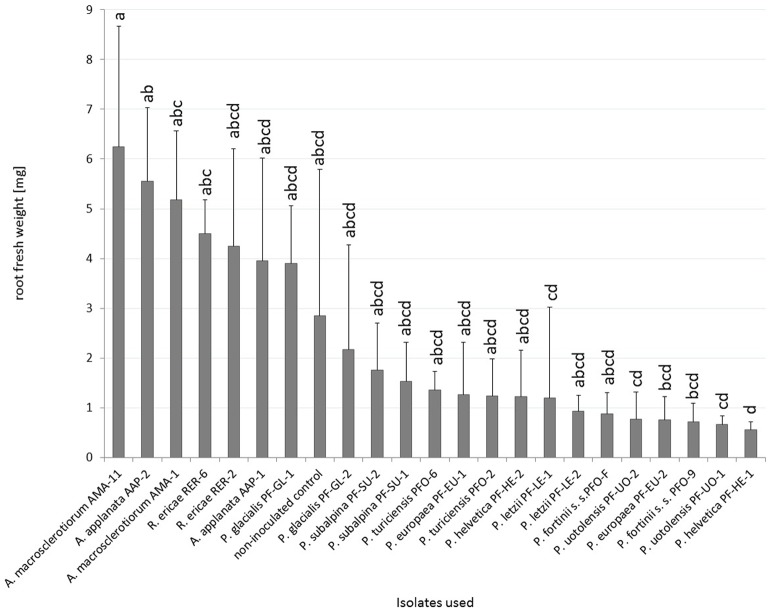
The effect of inoculation on blueberry fresh root weight in Experiment 2. Blueberry seedlings were inoculated by 8 PAC species, 2 species related to PAC and *Rhizoscyphus ericae* as a positive control, two strains per each species. Blueberry seedlings were grown in a peat-based substrate for 3.5 months under *in vitro* conditions. The presented data are means of 6 replicates ± standard error of mean. Different letters above the columns indicate significant differences according to the non-parametric Kruskal-Wallis test followed by the multiple-comparison z-value test.

With respect to the influence of the inoculation on blueberry fresh root weight, the inoculated plants did not differ from the non-inoculated control (Fig 5). Blueberries inoculated with *R*. *ericae* RER-6 had significantly higher fresh root biomass than those inoculated with *P*. *helvetica* PF-HE-1 but no other statistically significant differences from mycorrrhizal control were detected. Blueberries inoculated with *A*. *applanata* AAP-2 had significantly higher fresh root biomass than *P*. *helvetica* PF-HE-1, *P*. *letzii* PF-LE-1 and both strains of *P*. *uotolensis* but no other statistically significant differences within PAC were found. Blueberries inoculated with *A*. *macrosclerotiorum* AMA-11 had higher fresh root biomass than six PAC strains (*P*. *europaea* PF-EU-2, *P*. *fortinii* s. s. PFO-9, *P*. *helvetica* PF-HE-1, *P*. *letzii* PF-LE-1 and both strains of *P*. *uotolensis*). *A*. *macrosclerotiorum* AMA-1 differed significantly only form *P*. *helvetica* PF-HE-1. The most prominent intraspecific variation was found in *P*. *subalpina* and *P*. *glacialis* although it was not statistically significant (Fig 5).

Shoot dry weight and fresh root weight were negatively correlated (R = -0.469; p = 0.000) with fungal colonization. However, there were no statistical differences among the strains.

### Experiment 3: *In vitro* inoculation of birch in a peat-agar medium

All inoculated seedlings possessed some intraradical fungal colonization. The percentage of colonized root sections was significantly higher in *A*. *applanata* AAP-1 and *A*. *macrosclerotiorum* AMA-1 than in *A*. *macrosclerotiorum* AMA-11 (Table 3). *A*. *macrosclerotiorum* did not form any EcM structures in birch roots but these were colonized intracellulary by loose hyphal loops and intercellulary by melanised running hyphae (Fig 2g). *A*. *applanata* formed intracellular microsclerotia (Fig 2h) as well as sclerotia on the root surface. *P*. *involutus* formed extraradical hyphal mantles and a Hartig net in the roots of all inoculated birch seedlings (Table 2). The control non-inoculated plants did not show any signs of fungal colonization.

**Table 3:** Colonization, root fresh weight and shoot dry weight of birch seedlings inoculated by two strains of *A*. *macrosclerotiorum*, one strain of *A*. *applanata* and one strain of *P*. *involutus* as a positive EcM control fungus in Experiment 3.

Inoculated fungi	Colonization [%]	Root fresh weight [mg]	Shoot dry weight [mg]	CO_2_ concentration [ppm]
A. *macrosclerotiorum* AMA-1	51.79 ±15.34 a	9.2 ± 3.8 ab	6.1 ± 1.0 a	350.71 ± 22.13 a
A. *macrosclerotiorum* AMA-11	18.75 ± 10.90 b	11.7 ± 2.8 a	5.3 ± 0.9 ab	255.71 ± 67.73 ab
A. *applanata* AAP-1	61.07 ± 14.06 a	11.5 ± 2.5 a	7.3 ± 2.7 a	265.57 ± 33.16 ab
P. *involutus* PIN-1	not measured	10.5 ± 5.2 ab	4.1 ± 1.7 ab	222.00 ± 54.42 b
non-inoculated	no colonization	3.2 ± 1.0 b	2.6 ± 0.7 b	224.86 ± 63.17 b

Plants were cultivated in a peat-agar medium in *in vitro* conditions for 6 months. Concentration of CO_2_ in the microcosms was measured three days prior harvest. The presented data are means of 6 replicates ± SE. Different letters indicate significant differences according to the non-parametrical Kruscal-Wallis test followed by a multiple-comparison z-value test.

The inoculation had significant influence on birch shoot dry weight; the plants inoculated with *A*. *applanata* AAP-1 and *A*. *macrosclerotiorum* AMA-1 had significantly higher shoot biomass than the control non-inoculated plants. Differences were also observed in fresh root weight; birch seedlings inoculated with both *A*. *macrosclerotiorum* strains had higher root biomass than the non-inoculated control plants (Table 3).

No significant differences were detected in O_2_ concentration measured in the microcosms. Microcosms with *A*. *macrosclerotiorum* AMA-1 had higher CO_2_ concentration in comparison with both positive mycorrhizal and negative non-inoculated controls (Table 3). No contaminations of the peat substrate were detected and all the inoculated fungi were viable at the end of the experiment.

### Culture dependent screening of DSE-like fungi in Ericaceae roots from a pine forest

The microscopic screening of Ericaceae roots collected in the pine forest showed that 871 from the 1000 screened rhizodermal cells were intracellularly colonized by fungal hyphae, mostly in a manner typical for ericoid mycorrhiza. About one third of the obtained isolates (i.e., 35 isolates) produced brownish to blackish colonies typical for DSE fungi, including *A*. *macrosclerotiorum*. However, according to sequence analyses in BLAST, all but one sequence belonged to *P*. *fortinii* s. l. and none of the sequences belonged to *A*. *macrosclerotiorum* (S1 Table).

### Culture independent screening of fungal root symbionts in Ericaceae roots from the pine forest

**Fig 6:**
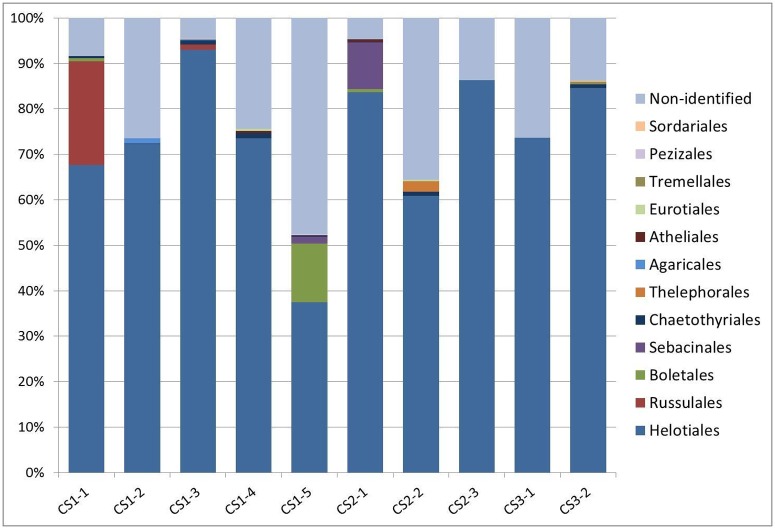
Relative abundances of the dominant fungal orders in Ericaceae roots detected by pyrosequencing. Tag-encoded pyrosequencing was performed with ten Ericaceae hair roots samples from three sites in NP České Švýcarsko (CS1—five samples, CS2—three samples, CS3—two samples) where the ectomycorrhizal morphotype *Pinirhiza sclerotina* formed by the DSE fungus related to PAC *Acephala macrosclerotiorum* was present. The obtained data were processed as described in Materials and Methods. OTUs with lower similarity and coverage than 88% were assigned as non-identified together with *incertae sedis* species. Orders less abundant than 0.1% were excluded from the figure.

On average we obtained 2184 fungal sequences per sample (ranging from 326 to 10076 per sample) and 37.9 OTUs per sample (ranging from 19 to 151); in total, we obtained 380 well-defined OTUs (S2 Table). All samples were dominated by Helotiales (Fig 6). The most abundant species across all localities (OTU 1) belonged to Helotiales, its abundance ranged from 82.15% to 5.59% (average 31.46%) (S2 Table) and its consensus sequence was most similar (94%) to an uncultured fungus sampled from a pine forest soil (GenBank acc. no. JX032276). The phylogenetic analysis placed this fungus in a sister clade (comprising only entries without scientific names) of a clade comprising *Claussenomyces*, *Collophora*, *Satchmopsis*, and some other fungi (S1 Fig). The second most abundant species across all localities (OTU 3) belonged to *P*. *fortinii* s. l. and its abundance ranged from 53.37% to 0.6% (average 18.76%). The third most abundant species across all localities (OTU 4) was present in all but one sample, its abundance ranged from 38.16% to 0.00% (average 10.65%) and its consensus sequence was identical to several uncultured fungi from different sources and locations (e.g., KF617263 from *Picea mariana* forest soil in Alaska, HM059043 from muskeg bog in Alaska or AB521974 from *Vaccinium* hair roots in Sweden). The phylogenetic analysis placed this fungus in the vicinity of many uncultured fungi from Chaetothyriales and Verrucariales (S2 Fig).

**Fig 7:**
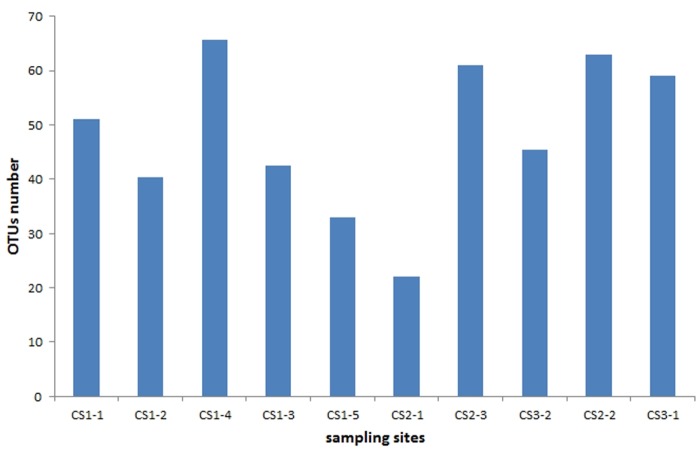
Values of Chao-1 index in equally resampled localities. The values of Chao-1 indexes for the respective localities are as follows: CS1-1 = 51, CS1-2 = 40.3, CS1-3 = 42.5, CS1-4 = 65.6, CS1-5 = 33, CS2-1 = 22, CS2-2 = 63, CS2-3 = 61, CS3-1 = 59 and CS3-2 = 45.5.

**Fig 8:**
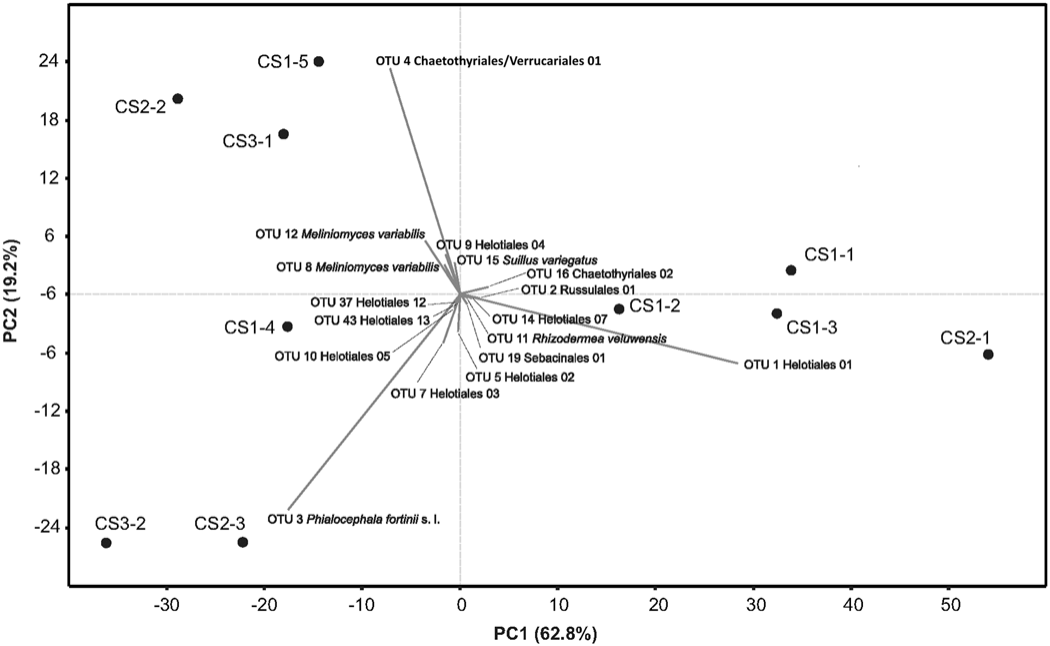
Principal component analysis of the relative abundance of OTUs. Only OTUs with component loadings for the first or second axis higher than 0.015 were visualized. For detailed information about the respective OTUs see S2 Table.

Based on Chao-1, the alpha diversity within samples varied from 22 to 66 OTUs with the most diverse sample being CS1-4 and the least diverse sample CS2-1 (Fig 7). In the PCA analysis, the first canonical axis explained 62.8% of the variability while the second axis explained 19.2% (Fig 8).

We did not obtain any sequences similar to *A*. *macrosclerotiorum* although the *Pinirhiza sclerotia* ectomycorrhizal morphotype was present in the pine roots in all screened samples.

## Discussion

### The DSE symbiotic potential in spruce and blueberry

Fungi related to *P*. *fortinii* are often isolated from mycorrhizal and non-mycorrhizal conifer roots and in re-synthesis experiments they frequently form pseudomycorrhizae sensu Melin [54] [4,55]. Although some authors reported that *P*. *fortinii* s. l. formed hyphal mantles and Hartig net in re-synthesis experiments with conifers, these structures were typically accompanied by intracellular hyphal colonization [28]. To our knowledge only two papers reported distinct ectomycorrhizal morphotypes formed by fungi related to *P*. *fortinii* s. l. [32,56]. Kaldorf *et al*. [56] reported that a relatively frequent black unbranched ectomycorrhizal morphotype with abundant emanating hyphae formed on hybrid aspen repeatedly yielded DNA of *P*. *fortinii* s. l. However, the authors did not use the respective fungus in a re-synthesis experiment and the amplified *P*. *fortinii* s. l. DNA might as well originate from endophytic hyphae [32]. In contrast, Münzenberger *et al*. [32] experimentally verified that *A*. *macrosclerotiorum* formed the ectomycorrhizal morphotype *P*. *sclerotia*. Here, under experimental conditions favorable for ectomycorrhiza formation, we showed that the tested strains representing all hitherto described PAC cryptic species failed to form ectomycorrhizae with spruce, in contrast to *A*. *macrosclerotiorum*. The latter species thus remains the only experimentally verified DSE forming a distinct ectomycorrhizal morphotype both under natural and artificial conditions. However, its abundance in ectomycorrhizal conifers is not clear [32,57] and the ecophysiological significance of *Pinirhiza sclerotia* needs to be further investigated.

Fungi related to *P*. *fortinii* s. l. are often isolated also from ericaceous hair roots and there are some reports showing that they may form intracellular hyphal coils in Ericaceae rhizodermis [30,31]. On the other hand, similar structures may be to some extent formed in Ericaceae hair roots also by soil saprophytic fungi [58]. Here, *A*. *applanata* and *A*. *macrosclerotiorum* formed intracellular hyphal coils in *Vaccinium* rhizodermal cells which strongly resembled those formed in ericoid mycorrhiza. In contrast to Vohník *et al*. [30] this colonization pattern was neither rare nor limited to only a few rhizodermal cells, reached colonization levels of the typical ericoid mycorrhizal fungus *R*. *ericae* and would be unambiguously considered as ericoid mycorrhiza if seen in naturally colonized roots. Additionally, the detected trend of increased dry shoot weight and fresh root weight connected with inoculation of blueberries with *A*. *applanata* and *A*. *macrosclerotiorum* (see below) suggests positive effects of these two DSE species on their host plants. However, this needs to be tested again under different experimental conditions and with more inoculation replicates.

*A*. *applanata* and *A*. *macrosclerotiorum* simultaneously formed intracellular hyphal coils resembling ericoid mycorrizae and typical DSE microsclerotia in the same hair roots. This parallels the observations made by Vohník and Albrechtová in roots of the European autochthonous *Rhododendron kotschyi* [33] and indeed suggests the existence of morphological continuum between ericoid mycorrhizal and DSE fungi.

### Host response to DSE colonization

Due to their high incidence in mycorrhizal roots, the effect of DSE on host plants has already been studied for more than a century. In his classical work, Melin [54] stated that the isolated DSE related to *P*. *fortinii* s. l. (*Mycelium radices atrovirens*) did not form ectomycorrhiza with conifers which was necessary for their normal growth; instead, they formed the harmful pseudomycorrhiza. Indeed, several other authors pointed out that DSE colonization in trees is harmful and its negative effects may be alleviated by ectomycorrhizal fungi [59–61].

Here we mainly focused on the growth response of ericaceous blueberries to inoculation with DSE. The colonization rate of the respective inoculated DSE strains significantly varied with negative correlation between the colonization rate and shoot dry weight, and root fresh weight. Although shoot dry weight of the plants inoculated with the typical ericoid mycorrhizal fungus *R*. *ericae* and the two ericoid mycorrhiza forming strains of *A*. *macrosclerotiorum* did not differ from the non-inoculated control it did differ from the plants inoculated with strains of *P*. *helvetica* and *P*. *uotolensis*. The effect of several PAC strains on the growth of blueberries was negative in comparison with those forming ericoid mycorrhizae with *R*. *ericae*. Although *R*. *ericae* and PAC fungi often coexist in the same ericaceous roots [62–71] it seems that these fungal guilds are functionally well separated. Despite their negative effect on Ericaceae in monoxenic *in vitro* experiments, fungi related to *P*. *fortinii* s. l. usually form a significant part of the Ericaceae mycoflora which was true also in this study. It is thus plausible to assume that ericoid mycorrhizal fungi may alleviate negative influence of DSE [15] similarly to ectomycorrhizal fungi in conifers. However, there are too few studies on Ericaceae for making any solid conclusion.

Despite that the tested DSE strains did not form ectomycorrhizae with birch, their presence significantly increased birch shoot and root biomass in comparison with non-inoculated control. In the case of *A*. *macrosclerotiorum* AMA-1, this non-mycorrhizal functioning may be at least partly attributed to the elevated CO_2_ concentration which stimulates net plant carbon gain from photosynthesis. Another possible mechanism may be mineralization of the cultivation substrate [23].

### Host specificity in DSE

The low to none host specificity of DSE [6,7] has been challenged after the division of P. *fortinii* s. l. into cryptic species, and description of two new DSE species, *A*. *macrosclerotiorum* and *P*. *glacialis*. Grünig and colleagues [65] investigated three subalpine forest sites in Switzerland with simultaneous presence of *P*. *abies* and *Vaccinium* spp. and reported that *A*. *applanata* preferred spruce roots while *P*. *subalpina* showed preference for Ericaceae; *P*. *glacialis* has been so far isolated only from Ericaceae hair roots and spruce needles, but not spruce roots [66]; and *A*. *macrosclerotiorum* formed ectomycorrhizae with pine but not with hybrid aspen [32]. Here we confirmed the ectomycorrhizal preference of *A*. *macrosclerotiorum* for conifers and extended the known range of its potential hosts for Norway spruce. On one hand, *A*. *macrosclerotiorum* has been isolated from spruce ectomycorrhizae already by Menkis *et al*. [57] few years before its formal taxonomic description; on the other hand it remains unclear whether the isolated *Phialocephala* 6/*Acephala* sp. 6 (conspecific with *A*. *macrosclerotiorum*, see http://unite.ut.ee/sh/SH213469.06FU) formed the respective ectomycorrhizae or lived inside them as an endophyte. The reason for the peculiar *A*. *macrosclerotiorum* mycorrhizal incompatibility with broadleaved trees remains unknown but may parallel host preferences of other (ecto-)mycorrhizal fungi. Another reason may be in the hypothesized higher conifer tolerance to DSE colonization: Ahlich and Sieber [67] and Grünig and colleagues [3] reported that DSE colonization densities tended to be lower in broadleaf trees than in conifers and suggested that conifers might better tolerate DSE in order to prevent infection by more serious fungal pathogens.

Despite that DSE often represent the majority of mycobionts isolated from ericaceous hair roots [10,63,64] we did not isolate or detect *A*. *macrosclerotiorum* in the Ericaceae roots at sites with significant presence of its ectomycorrhizal morphotype in neighboring pines. Such absence is intriguing given the apparent ability of *A*. *macrosclerotiorum* to form intracellular hyphal coils morphologically identical to ericoid mycorrhiza in Ericaceae rhizodermis (see Results). However, this species has not yet been detected in Ericaceae roots which suggests that the *A*. *macrosclerotiorum in vitro* ericoid mycorrhizal potential does not need to be realized under natural conditions. Similar scenario might hold true also for *A*. *applanata* which formed ericoid mycorrhizae *in vitro* but has not yet been reported in Ericaceae roots.

### Possible role of DSE in common mycorrhizal networks

The above mentioned low DSE host specificity along with the ability to simultaneously colonize plants with different mycorrhizal preferences [6,7] opens the potential to link neighboring plants with common mycelia. Such a scenario has been hypothesized for ectomycorrhizal plants and their ericaceous undergrowth because these plant guilds can form ectomycorrhizal and ericoid mycorrhizal symbioses with the same members of the *R*. *ericae* aggregate (REA) under *in vitro* conditions [34,68]. However, Kohout *et al*. [41] showed that the potentially shared REA mycobiont, *Meliniomyces bicolor*, was in fact suppressed when conifers and Ericaceae were grown together in an open air pot experiment. Similarly, the abundance of REA members in Ericaceae roots was significantly lower at sites with ectomycorrhizal tree dominants when compared to sites where the dominant trees preferred arbuscular mycorrhizal fungi [10]. Congruently, we found that *in vitro*, *A*. *macrosclerotiorum* was able to form ectomycorrhiza and ericoid mycorrhiza with spruce and blueberry, respectively, but this species was not detected in Ericaceae roots at natural localities with regular presence of the *P*. *sclerotia* morphotype which is formed by *A*. *macrosclerotiorum*. Our study thus provides another observation which suggests that mycorrhizal links between ectomycorrhizal conifers and ericoid mycorrhizal Ericaceae are under natural conditions rare to absent and perhaps even inhibited [10,41].

### Diversity of Ericaceae root mycobionts

In comparison with arbuscular mycorrhizal and ectomycorrhizal plants, the diversity of Ericaceae root mycobionts is tackled relatively scarcely. There are some reports from Argentina [10], Australia [69,70], Canada [64,71], China [72], Japan [73], Scandinavia [39,62], UK [74,75] and USA [63] but these are relatively scarce and studies from other regions, including central Europe, are missing. Although it is premature to speculate on the global diversity of Ericaceae mycobionts it roughly seems that some REA members (*M*. *variabilis*, *R*. *ericae*) together with *Oidiodendron maius* are dominant ErM fungi in temperate, boreal and subarctic Eurasia while Sebacinaceae and other mycobionts possibly form the dominant part of Ericaceae mycobionts in the North America and Southern Hemisphere [10]. In contrast, the Ericaceae hair roots investigated in this study were mostly free of the prominent ErM fungi, including Sebacinaceae, and their dominant mycobionts, except *P*. *fortinii* s. l., belonged to hitherto undescribed fungi from Helotiales and Chaetothyriales/Verrucariales [76]. The significance of this finding remains obscure yet it has to be kept in mind that most of the screened hair roots possessed ericoid mycorrhizae, similarly to [71]. We obviously cannot prove that the dominant mycobionts detected in this study using pyrosequencing indeed formed the observed intracellular hyphal structures. This could be achieved by a method targeting single host cells, e.g., laser capture microdissection followed by DNA isolation and PCR, which however has not yet been applied on ericoid mycorrhizae. The ITS sequence of the most common OTU 1 (Helotiales 01) clustered with many uncultured fungi and it is thus plausible to assume that OTU 1 is a non-cultivable species; on the other hand, two similar ITS sequences (FM172779 and HM208736) belong to isolates obtained from ericaceous roots. This situation emphasizes the importance of cultivation-based methods which may, when followed by re-synthesis experiments and experiments tracking the bi-directional mycorrhizal nutrient transfer, confirm the putative ericoid mycorrhizal status of dominant but hitherto undescribed Ericaceae mycobionts, especially from areas which have not yet been investigated.

The total diversity of Ericaceae mycobionts was relatively high but the three most abundant OTUs, Helotiales 01, *Phialocephala fortinii* s. l. and Chaetothyriales/Verrucariales 01, comprised over 60% of the total mycobiont abundance. These three OTUs showed strong preference for certain experimental sub-sites where they dominated the respective mycobiont community while being infrequent to absent at other sub-sites. *M*. *variabilis* (OTU 12), the most abundant typical ErM fungus detected in this study (yet with only 4.04% of the total mycobiont abundance) represented the most abundant mycobiont at one sub-site (23.01%) while being totally absent at other four sub-sites and nearly absent (< 0.5%) at another three sub-sites. This suggests that the community composition of ericaceous mycobionts may be significantly different even within sites with no apparent ecological gradients [38,64,74].

Interestingly, a minor part of the mycobiont spectra was formed by basidiomycetous EcM fungi from Agaricales, Boletales, Russulales and Thelephorales. The presence of EcM basidiomycete DNA in Ericaceae roots has been already reported [63,74] and non-sebacinaceous basidiomycetes apparently have the ability to colonize Ericaceae rhizodermis [10,33] but their functional role is virtually unknown. Some may intracellularly colonize healthy ericaceous roots under *in vitro* conditions but the resulting colonization pattern does not resemble typical ericoid mycorrhizae [35,77] and it is plausible that under natural conditions, these mycobionts colonize senescent or moribund hair root cells and act as opportunistic saprobes rather than true ErM fungi.

Sebacinaceae, a group of ubiquitous heterobasidiomycetous endophytes and mycorrhizal fungi [78] were proposed as common ericoid mycorrhizal fungi worldwide, based on culture-independent methods [79]. Here we detected Sebacinaceae sequences only at two sub-sites out of ten with the total abundance of 1.18%. This was a surprising finding given the absence of other common ericoid mycorrhizal fungi and the fact that Sebacinaceae often represent a major component of Ericaceae mycobiont communities [71,80]. To our knowledge, a successful isolation of a sebacinaceous strain from Ericaceae hair roots has been reported only once [35] and an experimental (re-synthetic) proof that these fungi indeed form ericoid mycorrhizae is still missing, although indirect evidence is strong [79]. The reason for the low incidence of Sebacinaceae (and other prominent ericoid mycorrhizal fungi) in this study is unknown and remains to be investigated.

## Conclusions

Although PAC fungi are often isolated from ectomycorrhizal and ericoid mycorrhizal roots, none of the tested PAC cryptic species except *A*. *applanata* formed typical mycorrhizal structures. Moreover, some of the tested PAC strains had negative influence on host biomass. Interspecific variability thus likely does not explain the inconsistency of the results obtained in the DSE research during past decades when differentiation of *P*. *fortinii*-related fungi to cryptic species was not possible. In contrast, it seems that the true reason is in variability of the many different combinations of particular DSE strains with particular host plants under particular growing conditions, as showed here and recently discussed by other authors [20,81]. On the other hand, some of the PAC close relatives apparently have ectomycorrhizal (*A*. *macrosclerotiorum*) and ericoid mycorrhizal (*A*. *macrosclerotiorum*, perhaps also *P*. *glacialis*) potential, which however does not need to be realized under natural conditions as suggested by the lack of *A*. *macrosclerotiorum* in Ericaceae hair roots in the pine fores with common occurrence of the *P*. *sclerotia* morphotype. To our knowledge, *A*. *macrosclerotiorum* is the only documented DSE fungus forming ectomycorrhizae. Interestingly, its ectomycorrhizal potential seems to be realized only in conifers (pine, spruce) but not in broadleaved plants (birch, poplar).

## Supporting Information

S1 Fig The taxonomic position of OTU 1. For details on the phylogenetic analyses see Materials and Methods.
(PDF)

S2 Fig The taxonomic position of OTU 4. For details on the phylogenetic analyses see Materials and Methods.
(PDF)

S1 Table The identity of dark septate endophytes obtained by culture-dependent approach. The table displays the three top GenBank hits to each sequence of the DSE isolates obtained from ericaceous hair roots in this study. Sequences derived from cultured isolates with scientific names were preferred.
(XLSX)

S2 Table The identity of the mycobionts detected by tag-encoded pyrosequencing. The table provides basic information on the 380 OTUs obtained in this study by tag-encoded pyrosequencing (see Materials and Methods, and Results). OTUs are in grey when the respective closest GenBank match had similarity <97% or there was no match in GenBank. The column "Closest match in GenBank" lists closest matches with scientific names.
(XLSX)
