# Supplementary figures and images for: The Potential of Dark Septate Endophytes to Form Root Symbioses with Ectomycorrhizal and Ericoid Mycorrhizal Middle European Forest Plants
Source: PLoS One. 2015 Apr 23;10(4):e0124752. doi: 10.1371/journal.pone.0124752 (PMC4408093; doi:10.1371/journal.pone.0124752)

Supplementary Figure 1: The taxonomic position of OTU 1

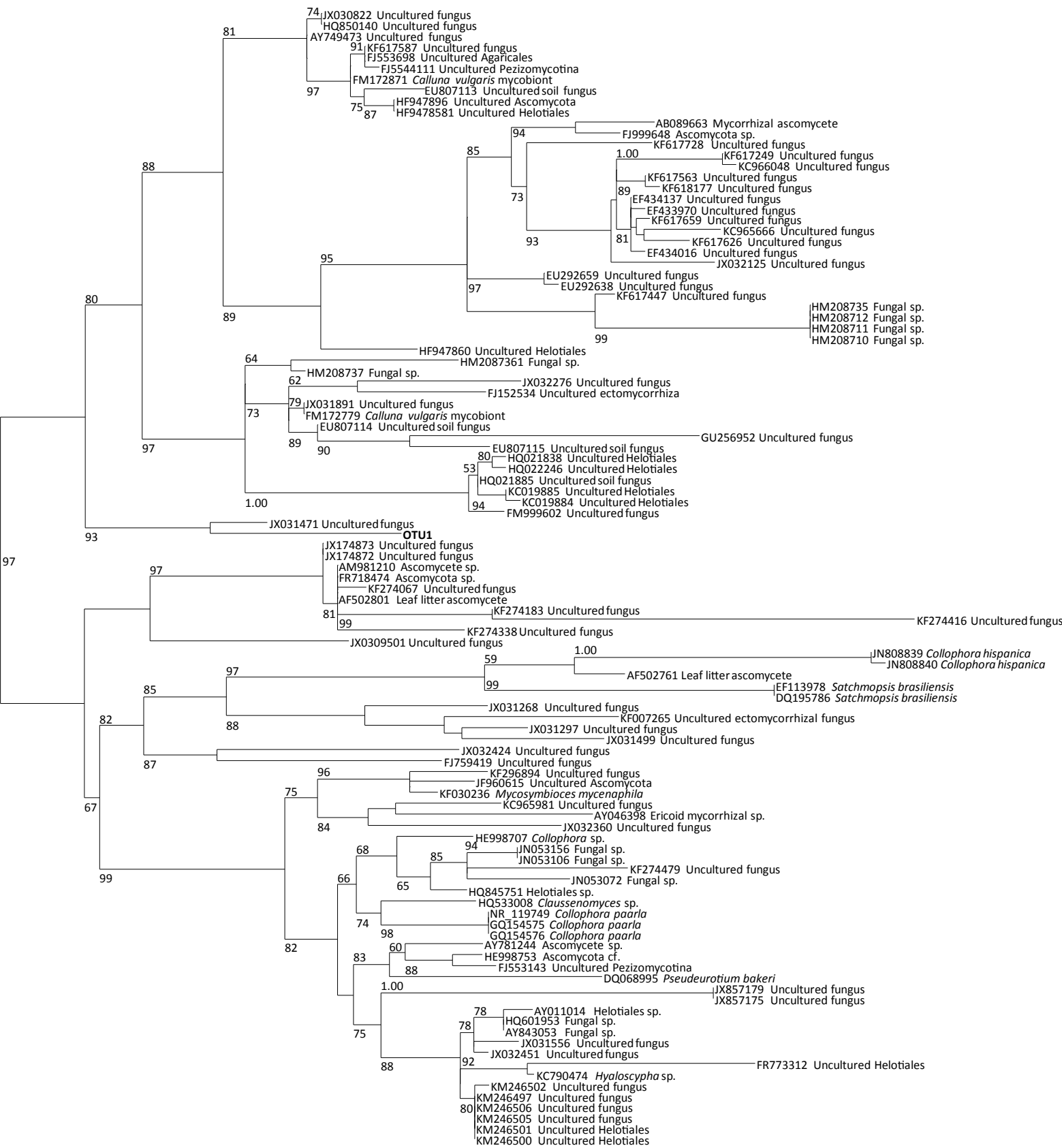

PhyML ln(L)= -3493.1 166 sites HKY85 4 rate classes

Supplement: S1 Fig — For details on the phylogenetic analyses see Materials and Methods. (PDF) [file pone.0124752.s001.pdf]

Supplementary Figure 2: The taxonomic position of OTU 4

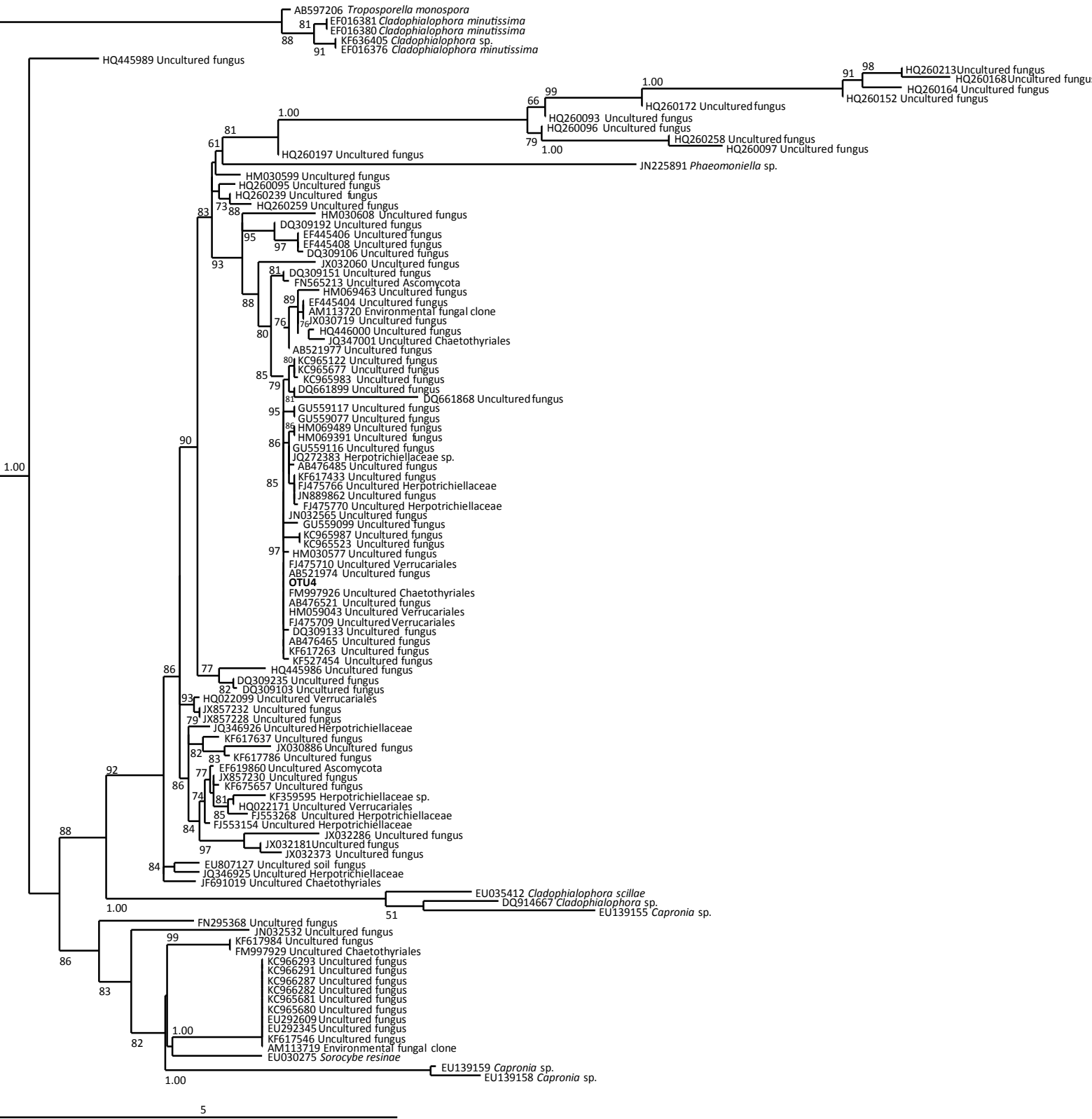

PhyML ln(L)= -3691.8 174 sites HKY85 4 rate classes

Supplement: S2 Fig — For details on the phylogenetic analyses see Materials and Methods. (PDF) [file pone.0124752.s002.pdf]
